# Efficacy of sacubitril/valsartan sodium combined with simvastatin in treating chronic heart failure with arrhythmia and its effects on IL-6, IL-8, and TNF-α

**DOI:** 10.5937/jomb0-59426

**Published:** 2026-01-06

**Authors:** Jingjing Li, Zhihua Wang

**Affiliations:** 1 Yancheng NO.1 People's Hospital, Department of Cardiovascular Medicine, Yancheng, China

**Keywords:** sacubitril/valsartan sodium, simvastatin, chronic heart failure, arrhythmia, clinical efficacy, inflammatory factors, sakubitril/valsartan natrijum, simvastatin, hronična srčana insuficijencija, aritmija, klinička efikasnost, inflamatorni faktori

## Abstract

**Background:**

To explore, in conjunction with clinical practice, the efficacy of different pharmacological treatment regimens for patients with chronic heart failure complicated by arrhythmia, and their effects on inflammatory factor levels.

**Methods:**

A total of 96 patients with chronic heart failure and arrhythmia treated at our hospital from June 2022 to January 2025 were selected and randomly assigned by envelope method into a combination therapy group and a simvastatin group. The simvastatin group received simvastatin monotherapy, while the combination group was treated with sacubitril/valsartan sodium plus simvastatin. Clinical efficacy was compared between the two groups. Cardiac function, inflammatory factors, arrhythmia episodes, blood lipid levels, and oxidative stress markers were assessed before and after treatment. Adverse reactions in both groups were also observed.

**Results:**

The overall effective rate in the combination therapy group (93.75%) was significantly higher than that in the simvastatin group (72.92%) (c2= 7.500, P=0.006). After treatment, the combination group exhibited higher LVEF and lower LVESD and LVEDD levels compared to the simvastatin group (P&lt; 0.05). Serum levels of inflammatory factors (IL-6, IL-8, TNF-a) were significantly lower in the combination group than in the simvastatin group after treatment (P&lt; 0.05). Both the duration and frequency of arrhythmia episodes were reduced in the combination group compared to the simvastatin group (P&lt; 0.05). Post-treatment, LDL-C, TG, and TC levels were lower, and HDL-C was higher in the combination group than in the simvastatin group (P&lt; 0.05). MDA and SOD levels were also lower in the combination group after treatment (P&lt; 0.05). The incidence of adverse reactions was lower in the combination group (3.33%) compared to the simvastatin group (22.92%) (c2 = 5.352, P= 0.021).

**Conclusions:**

For patients with chronic heart failure and arrhythmia, combined therapy with sacubitril/valsartan sodium and simvastatin demonstrates significant efficacy. It can alleviate inflammatory responses, improve cardiac function, reduce the frequency and duration of arrhythmia episodes, optimize lipid profiles and stress responses, and decrease adverse reactions. This approach is worthy of further clinical promotion.

## Abstract (sr)

**Uvod:**

Cilj je bio da se istraži u kombinaciji sa kliničkom praksom, efikasnost različitih farmakoloških režima lečenja pacijenata sa hroničnom srčanom insuficijencijom komplikovanom aritmijom, i njihov uticaj na nivoe inflamatornih faktora.

**Metode:**

Ukupno 96 pacijenata sa hroničnom srčanom insuficijencijom i aritmijom lečenih u našoj bolnici od juna 2022. do januara 2025. godine odabrano je i nasumično raspoređeno metodom envelope u grupu kombinovane terapije i grupu sa simvastatinom. Grupa sa simvastatinom je primala monoterapiju simvastatinom, dok je kombinovana grupa lečena sakubitrilom/valsartanom natrijumom plus simvastatin. Klinička efikasnost je upoređena između dve grupe. Srčana funkcija, inflamatorni faktori, epizode aritmije, nivoi lipida u krvi i markeri oksidativnog stresa procenjeni su pre i posle lečenja. Takođe su primećene neželjene reakcije u obe grupe.

**Rezultati:**

Ukupna stopa efikasnosti u grupi kombinovane terapije (93,75%) bila je značajno veća nego u grupi simvastatina (72,92%) (c2= 7,500, P= 0,006). Nakon tretmana, kombinovana grupa je pokazala viši LVEF i niže nivoe LVEDS i LVEDD u poređenju sa grupom simvastatina (P&lt; 0,05). Serumski nivoi inflamatornih faktora (IL-6, IL-8, TNF-a) bili su značajno niži u kombinovanoj grupi nego u grupi simvastatina nakon tretmana (P&lt; 0,05). I trajanje i učestalost epizoda aritmije smanjeni su u kombinovanoj grupi u poređenju sa grupom simvastatina (P&lt; 0,05). Nakon tretmana, nivoi LDL-C, TG i TC bili su niži, a HDL-C je bio viši u kombinovanoj grupi nego u grupi simvastatina (P&lt; 0,05). Nivoi MDA i SOD takođe su bili niži u kombinovanoj grupi nakon tretmana (P&lt; 0,05). Učestalost neželjenih reakcija bila je niža u grupi sa kombinovanom terapijom (3,33%) u poređenju sa grupom sa simvastatinom (22,92%) (c2 = 5,352, P= 0,021).

**Zaključak:**

Kod pacijenata sa hroničnom srčanom insuficijencijom i aritmijom, kombinovana terapija sakubitrilom/valsartanom natrijum i simvastatinom pokazuje značajnu efikasnost. Može ublažiti inflamatorne reakcije, poboljšati srčanu funkciju, smanjiti učestalost i trajanje epizoda aritmije, optimizovati lipidne profile i reakcije na stres, i smanjiti neželjene reakcije. Ovaj pristup je vredan dalje kliničke promocije.

## Introduction

Chronic heart failure with concomitant arrhythmia refers to patients who, on the basis of chronic heart failure, also present with cardiac arrhythmias [1]. Chronic heart failure is a clinical syndrome representing the advanced stage of various heart diseases, in which the heart is unable to pump sufficient blood to meet the body's needs [2] [3]. Arrhythmia is characterized by abnormal electrical activity of the heart, resulting in changes in heart rhythm or rate. With an aging population and rising incidence of cardiovascular diseases, the prevalence of chronic heart failure complicated by arrhythmia is increasing year by year, and these patients face higher rates of mortality and rehospitalization. The main treatment approach is pharmacotherapy, using antiarrhythmic drugs to control heart rhythm, along with diuretics, angiotensinconverting enzyme inhibitors, and other medications to improve cardiac function. Sacubitril/valsartan sodium is a combination cardiovascular medication classified as an angiotensin receptor-neprilysin inhibitor (ARNI), consisting of sacubitril (a neprilysin inhibitor prodrug) and valsartan (an angiotensin II receptor blocker) [4]. Sacubitril inhibits neprilysin, increasing natriuretic peptide levels to promote vasodilation and sodium excretion, while valsartan blocks angiotensin II receptors, suppressing the renin-angiotensin system. This combination is indicated for patients with chronic heart failure with reduced ejection fraction and can reduce the risk of cardiovascular death and heart failure hospitalization. Simvastatin is a lipid-lowering agent belonging to the class of HMG-CoA reductase inhibitors, which suppresses the activity of the key enzyme HMG-CoA reductase in cholesterol synthesis, thereby reducing endogenous cholesterol production [5] [6]. This study investigates the efficacy of different pharmacological regimens in treating chronic heart failure complicated by arrhythmia, as reported below.

## Materials and methods

### General information

A total of 96 patients with chronic heart failure complicated by arrhythmia who were treated at our hospital between June 2022 and January 2025 were selected for this study. Using the envelope randomization method, patients were divided into a combination therapy group and a simvastatin group, with 48 cases in each group. There were no significant differences in baseline characteristics between the two groups (P > 0.05). See Table 1.

**Table 1:** Comparison of baseline data between the two groups (x̄±s, n(%)).

Group	n	Gender		Age<br>(year)	Disease Duration<br>(years)
		Male	Female		
Combination Therapy Group	48	27	21	70.50±8.43	5.73±1.43
Simvastatin Group	48	25	23	70.32±8 .32	5.69±1.59
* t/χ^2^*			0.168	0.105	0.130
* P *			0.682	0.917	0.897

### Inclusion and exclusion criteria

### Inclusion criteria

(1) Patients meet the relevant diagnostic criteria [6]; (2) Arrhythmia is confirmed by echocardiography; (3) No recent history of anti-infective or immunotherapy; (4) Informed consent is obtained from both patients and their families.

### Exclusion criteria

(1) Presence of malignant tumors; (2) Presence of hematological or immune system diseases; (3) Allergic reactions to the medications used in this study; (4) Pregnant or lactating women.

### Study methods

The simvastatin group received simvastatin (manufactured by Zhejiang Jingxin Pharmaceutical Co., Ltd., National Drug Approval No. H20000009), administered orally at 20 mg per dose, once daily. The combination therapy group received additional sacubitril/valsartan sodium (manufactured by Changzhou Pharmaceutical Factory Co., Ltd., National Drug Approval No. H20243657), administered orally at 100 mg per dose, once daily. Both groups underwent continuous treatment for three months.

### Criteria for efficacy evaluation

Markedly effective: Clinical symptoms such as dizziness and chest tightness disappeared, and the frequency of arrhythmia episodes decreased by more than 90%;

Effective: Clinical symptoms improved, and arrhythmia episodes decreased by 50%-90%;

Ineffective: No improvement or worsening of symptoms, and no change in the frequency of arrhythmia episodes.

### Observation indicators

(1) Cardiac color Doppler ultrasound was performed before and after treatment to assess cardiac function in both groups, including left ventricular ejection fraction (LVEF), left ventricular end-systolic diameter (LVESD), and left ventricular end-diastolic diameter (LVEDD);

(2) Levels of inflammatory markers were measured before and after treatment using enzyme-linked immunosorbent assay (ELISA), including interleukin-6 (IL-6), interleukin-8 (IL-8), and tumor necrosis factor-alpha (TNF-α);

(3) The duration and frequency of arrhythmia episodes before and after treatment were recorded for both groups;

(4) Blood lipid levels were measured before and after treatment using a fully automated biochemical analyzer, including total cholesterol (TC), triglycerides (TG), high-density lipoprotein cholesterol (HDL-C), and low-density lipoprotein cholesterol (LDL-C);

(5) Stress response was evaluated before and after treatment in both groups, including malondialdehyde (MDA) and superoxide dismutase (SOD) levels;

(6) Incidence of adverse reactions in both groups.

### Statistical analysis

Statistical analysis was performed using Statistic Package for Social Science (SPSS) 26.0 (IBM, Armonk, NY, USA). Categorical data were expressed as n or %, and the chi-square test (χ^2^) was used. Continuous data were expressed as mean ± standard deviation (x̄±s), and the t-test was used. A P value < 0.05 was considered statistically significant.

## Results

### Comparison of clinical efficacy between the combination therapy group and the simvastatin group

The overall effective rate in the combination therapy group was 93.75%, which was higher than that of the simvastatin group at 72.92% (χ^2^ = 7.500, P = 0.006). See Table 2.

**Table 2:** Comparison of clinical efficacy between the combination therapy group and the simvastatin group (n, (%)).

Group	n	Markedly Effective	Effective	Ineffective	Total Effective (n (%))
Combination	48	29 (60.42%)	16 (33.33%)	3 (6.25%)	45 (93.75%)
Simvastatin Group	48	14 (29.17%)	21 (43.75%)	13 (27.08%)	35 (72.92%)
* χ^2^*					7.5
* P *					0.006

### Comparison of cardiac function indicators before and after treatment between the combination therapy group and the simvastatin group

Compared with the Simvastatin group, patients in the Combination Therapy group had higher LVEF levels and lower LVESD and LVEDD levels after treatment (P<0.05). See Table 3.

**Table 3:** Comparison of cardiac function indicators before and after treatment between the combination therapy group and the simvastatin group (x̄±s). Note: Compared with before treatment, *P<0.05.

Group	n	LVEF (%)		LVESD (mm)		LVEDD (mm)	
		Before	After	Before	After	Before	After
Combination Therapy	48	46.07±4.64	61.35±6.28*	65.42±6.52	47.53±4.70*	52.21±5.16	43.26±4.47*
Simvastatin Group	48	46.21±4.15	52.17±5.12*	65.39±6.71	54.40±5.02*	52.28±5.27	48.15±5.08*
* t *		0.156	7.849	0.022	6.921	0.066	5.007
* P *		0.876	0.000	0.982	0.000	0.948	0.000

### Comparison of serum inflammatory factor levels before and after treatment between the combination therapy group and the simvastatin group

Compared with the simvastatin group, patients in the combination therapy group showed significantly lower levels of IL-6, IL-8, and TNF-α after treatment (P<0.05). See Table 4.

**Table 4:** Comparison of serum inflammatory factor levels before and after treatment between the combination therapy group and the simvastatin group (x̄±s).

Group	n	IL-6 (pg/mL		IL-8 (pg/mL		TNF-α (ng/mL)	
		Before	After	Before	After	Before	After
Combination Therapy	53	23.64±2.16	8.38±1.59*	21.81±2.04	10.07±1.25*	3.31±0.45	1.61±0.23*
Simvastatin Group	53	23.68±2.12	13.60±1.42*	21.75±2.22	15.79±1.76*	3.36±0.47	2.52±0.35*
* t *		0.092	16.965	0.138	18.358	0.532	15.054
P		0.927	0.000	0.891	0.000	0.596	0.000

### Comparison of arrhythmia episodes before and after treatment between the combination therapy group and the simvastatin group

Compared with the simvastatin group, patients in the combination therapy group had shorter durations and lower frequencies of arrhythmia episodes after treatment (P<0.05). See Table 5.

**Table 5:** Comparison of arrhythmia episodes before and after treatment between the combination therapy group and the simvastatin group (x̄±s). Note: Compared with before treatment, *P<0.05.

Group	n	Duration (min)		Attack Frequency (times/month)	
		Before	After	Before	After
Combination Therapy	48	10.52±1.50	5.15±0.59*	5.05±0.59	1.36±0.32*
Simvastatin Group	48	10.43±1.55	7.58±0.83*	5.11±0.56	2.88±0.43*
* t *		0.289	16.532	0.511	19.647
* P *		0.773	0.000	0.611	0.000

### Comparison of blood lipid levels before and after treatment between the combination therapy group and the simvastatin group

Compared with the simvastatin group, patients in the combination therapy group had lower levels of LDL-C, TG, and TC, and higher levels of HDL-C after treatment (P<0.05). See Table 6.

**Table 6:** Comparison of blood lipid levels before and after treatment in the combination therapy group and the simvastatin group (x̄±s, mmol/L). Note: Compared with before treatment, *P<0.05.

Group	n	HDL-C		LDL-C		TG		TC	
		Before	After	Before	After	Before	After	Before	After
Combination<br>Therapy	48	1 .82 ± 0 .26	2 .57 ± 0 .47 *	3 .83 ± 0 .38	2 .04 ± 0 .27 *	6 .21 ± 0 .75	3 .06 ± 0 .38 *	6 .33 ± 0 .87	5 .04 ± 0 .56 *
Simvastatin<br>Group	48	1 .80 ± 0 .23	2 .04 ± 0 .34 *	3 .86 ± 0 .41	3 .12 ± 0 .35 *	6 .25 ± 0 .72	* K- LO О +1 CO	6 .37 ± 0 .76	5 .86 ± 0 .62 *
*t*		0 .399	6 .330	0 .372	16 .927	0 .267	15 .469	0 .240	6 .800
*P*		0.691	0 .000	0.711	0 .000	0 .790	0 .000	0.811	0 .000

### Comparison of stress response indicators before and after treatment between the combination therapy group and the simvastatin group

Compared with the simvastatin group, the combination therapy group showed significantly lower MDA and SOD levels after treatment (P<0.05). See Table 7.

**Table 7:** Comparison of stress response indicator levels before and after treatment between the combination therapy group and the simvastatin group (x̄±s).

Group	n	MDA (μmol/L)		SOD (KU/L)	
		Before	After	Before	After
Combination Therapy	48	4.82±0.55	2.12±0.18*	2.81±0.35	2.03±0.16*
Simvastatin Group	48	4.89±0.52	2.95±0.35*	2.87±0.39	2.59±0.27*
* t *		0.641	14.611	0.793	12.362
* P *		0.523	0.000	0.430	0.000

### Comparison of adverse reactions between the combination therapy group and the simvastatin group

The incidence of adverse reactions in the combination therapy group was 3.33%, which was lower than that in the simvastatin group (22.92%) (χ2 = 5.352, P=0.021). See Table 8.

**Table 8:** Comparison of the incidence of adverse reactions between the combination therapy group and the simvastatin group (n, (%)).

Group	n	Nausea and vomiting	Hypotension	Fever	Liver and kidney <br>injury	Overall incidence
Combination <br>Therapy	48	1(2.08)	1(2.08)	0(0.00)	1(2.08)	3(6.25 )
Simvastatin Group	48	3(6.25)	3(6.25)	2(4.17)	3(6.25)	11(22.92)
*t *						5.352
* P *						0.021

## Discussion

The pathogenesis of chronic heart failure complicated by arrhythmia is complex. In chronic heart failure, structural and functional changes in the heart lead to myocardial remodeling, resulting in abnormal electrophysiological properties of cardiomyocytes that predispose to re-entry excitation and trigger arrhythmias [7]. Additionally, heart failure increases intracardiac pressure and volume load, leading to myocardial ischemia and hypoxia, which cause metabolic disturbances in cardiomyocytes and dysfunction of ion channels—including sodium, potassium, and calcium channels—thus affecting the depolarization and repolarization processes and inducing arrhythmias [8]. Furthermore, activation of the neuroendocrine system, such as sympathetic nervous system excitation and hyperactivity of the renin-angiotensin-aldosterone system, can further exacerbate myocardial electrical instability, promoting the onset of arrhythmias [9].

Simvastatin exerts multiple mechanisms in treating chronic heart failure with arrhythmia. From the perspective of improving myocardial remodeling, myocardial remodeling in chronic heart failure forms a key substrate for arrhythmia development [10]. As a 3-hydroxy-3-methylglutaryl-coenzyme A reductase inhibitor, simvastatin can suppress inflammatory responses, reduce the release of pro-inflammatory cytokines such as tumor necrosis factor-alpha and interleukin-6, alleviate myocardial inflammatory injury, and thereby inhibit myocardial remodeling. This leads to stabilization of the electrophysiological properties of cardiomyocytes and reduces the risk of arrhythmias [11] [12]. Regarding ion channel modulation, simvastatin can affect the function of ion channels on the cardiomyocyte membrane. It has the ability to stabilize membrane potential and regulate the transmembrane transport of sodium, potassium, and calcium ions, making the depolarization and repolarization processes in cardiomyocytes more regular and decreasing arrhythmias caused by ion channel abnormalities [13]. From the standpoint of antioxidative stress, patients with chronic heart failure often experience elevated oxidative stress, which can damage cardiomyocytes. Simvastatin has antioxidant properties, can scavenge oxygen free radicals, reduce oxidative stress-induced myocardial cell injury, protect the normal structure and function of cardiomyocytes, and maintain normal cardiac electrical activity [14]. In addition, simvastatin can improve cardiac autonomic nervous function by regulating the balance between the sympathetic and parasympathetic nervous systems, reducing sympathetic overactivity, thus decreasing susceptibility to arrhythmias and playing a therapeutic role in chronic heart failure with arrhythmias [15]. However, there are certain limitations to simvastatin in this context. Patient responses to the drug vary, and due to factors such as genetic polymorphism, some individuals may have reduced sensitivity to simvastatin, resulting in suboptimal lipid regulation and cardiac function improvement. Moreover, the adverse effects of simvastatin restrict its clinical use— it may cause hepatotoxicity with elevated transaminase levels and can induce myotoxicity manifesting as myalgia or muscle weakness; in severe cases, it may even lead to rhabdomyolysis. Therefore, simvastatin alone may not effectively control the condition, and combination therapy with other medications or non-pharmacological interventions may be necessary [16].

Although the changes in LVESD (left ventricular end-systolic diameter), LVEDD (left ventricular end-diastolic diameter), MDA (malondialdehyde), and SOD (superoxide dismutase) were statistically significant, the magnitude of these changes should also be evaluated in the context of clinical relevance.

Modest reductions in LVESD and LVEDD have been associated with improved cardiac remodeling and potential prognostic benefit in chronic heart failure patients. However, the degree of change reported in this study may not translate into meaningful improvements in symptoms or long-term outcomes without longer follow-up.

Similarly, changes in oxidative stress markers (MDA and SOD) indicate a possible shift in the oxidative balance, but their direct impact on patient-centric clinical outcomes remains to be fully elucidated and warrants further investigation in larger and longer-term studies.

Sacubitril/valsartan sodium treats chronic heart failure with concomitant arrhythmia through multiple mechanisms. From the perspective of improving cardiac function, it is a compound preparation: valsartan is an angiotensin II receptor antagonist that blocks the binding of angiotensin II to its receptor, dilates blood vessels, reduces peripheral vascular resistance, and decreases cardiac afterload; at the same time, it reduces aldosterone secretion, decreases water and sodium retention, lowers cardiac preload, and improves cardiac hemodynamics, thereby enhancing cardiac function [17]. As cardiac function improves, the electrical stability of the myocardium increases, which can reduce the incidence of arrhythmias. Sacubitril is a neprilysin inhibitor with diuretic, natriuretic, vasodilatory, and sympathetic nervous system inhibitory effects. By increasing the activity of natriuretic peptides, it further alleviates cardiac volume and pressure load, improves myocardial perfusion and oxygen supply, stabilizes the electrophysiological characteristics of myocardial cells, and decreases susceptibility to arrhythmias [18]. Additionally, sacubi- tril/valsartan sodium can inhibit myocardial remodeling. In chronic heart failure, structural and functional changes in the myocardium form an important basis for the development of arrhythmias [19]. This medication can regulate the neuroendocrine system, inhibit myocardial fibrosis and hypertrophy, and help normalize myocardial structure and function, thereby reducing the risk of arrhythmias.

The combination of sacubitril/valsartan sodium and simvastatin offers significant advantages in treating chronic heart failure with arrhythmia [20]. In terms of improving cardiac function, the two drugs have strong synergistic effects. Valsartan in sacubitril/valsartan sodium blocks angiotensin II receptors, dilates blood vessels, and reduces cardiac load; sacubitril inhibits neprilysin, enhances the effects of natriuretic peptides, further relieves cardiac burden, and improves hemodynamics. Simvastatin can lower cholesterol, improve endothelial function, reduce lipid deposition, and decrease blood viscosity, thereby improving myocardial perfusion [21]. The combination provides a more comprehensive and effective enhancement of cardiac function. From the antiarrhythmic perspective, sacubitril/valsartan sodium stabilizes myocardial electrical activity, inhibits myocardial remodeling, and reduces arrhythmia incidence. Simvastatin modulates ion channels and oxidative stress, maintaining the normal electrophysiological characteristics of myocardial cells. Combined therapy can reduce both the frequency and severity of arrhythmia episodes through multiple mechanisms. Furthermore, this combination therapy has a favorable safety profile; with appropriate dosing, it can exert therapeutic effects while reducing the adverse reactions that may result from high doses of a single drug, thereby providing a more optimized treatment strategy for patients with chronic heart failure and arrhythmia [22].

The results of this study showed that, compared with the simvastatin group, the total therapeutic efficacy was higher in the combination treatment group, indicating that combining sacubitril/valsartan sodium can improve clinical efficacy. After treatment, the combination group had higher LVEF and lower LVESD and LVEDD levels than the simvastatin group, suggesting improved cardiac function [23]. Serum inflammatory markers such as IL-6, IL-8, and TNF-α were lower in the combination group, indicating reduced inflammatory response. The duration and frequency of arrhythmia episodes were also lower in the combination group, suggesting that sacubitril/valsartan sodium can reduce the frequency and duration of arrhythmias [24]. Post-treatment, LDL-C, TG, and TC levels were lower and HDL-C was higher in the combination group, indicating improved lipid profiles. The combination group also had lower MDA and SOD levels, suggesting reduced oxidative stress. The incidence of adverse reactions was lower in the combination group, indicating higher safety [25]. These results are consistent with the findings of Wang Yu, Yang Kai, and others.

This study has several limitations. First, it was conducted as a single-center retrospective analysis, which may introduce selection bias and limit the generalizability of the findings to broader patient populations. The retrospective nature of the study also restricts the ability to establish causal relationships between interventions and outcomes. Second, the sample size was relatively small, with only 48 patients in each group, which may reduce the statistical power and increase the risk of type II errors. Therefore, the results should be interpreted with caution. To address these limitations, future research should include multicenter, prospective studies with larger sample sizes to further validate the efficacy and safety of sacubitril/valsartan sodium combined with simvastatin in patients with chronic heart failure and arrhythmia, and to enhance the generalizability and robustness of the findings.

## Conclusion

In summary, for patients with chronic heart failure and arrhythmia, the addition of sacubitril/valsartan sodium to simvastatin therapy yields significant benefits. It can reduce inflammatory responses, improve cardiac function, decrease the frequency and duration of arrhythmia episodes, improve lipid and stress responses, and lower the incidence of adverse reactions. This approach is worthy of further clinical promotion and application.

## Dodatak

### Conflict of interest statement

All the authors declare that they have no conflict of interest in this work.
